# Research on Hot Corrosion Behavior of DZ40M and K452 Superalloys in NaCl Molten Salt

**DOI:** 10.3390/ma15041512

**Published:** 2022-02-17

**Authors:** Lei Wan, Zeyu Zhang, Wenquan Wang, Yunpeng Xue, Jubao Shen, Tao Sun, Haiou Sun

**Affiliations:** 1 College of Power and Energy Engineering, Harbin Engineering University, Harbin 150001, China; wanlei1981@hrbeu.edu.cn (L.W.); hrbeujfzzy@163.com (Z.Z.); HEU_SUN@163.com (H.S.); 2 Shenyang National Laboratory for Materials Science, Northeastern University, Shenyang 110819, China; 18840699775@163.com (W.W.); 1900587@stu.neu.edu.cn (J.S.); 3 AECC Shenyang Liming Aero-Engine Co., Ltd., Shenyang 110043, China; XueYunPeng2022@163.com

**Keywords:** DZ40M, K452, NaCl molten salt, hot corrosion

## Abstract

The corrosion of cobalt-based DZ40M and nickel-based K452 superalloy at 900 °C was investigated by NaCl salt coating. Accordingly, the differences in hot corrosion behavior were analyzed considering the development methods and elementary composition by comparing the two alloys’ failure. Then, the corrosion mechanism induced by NaCl was proposed by comparing oxidation and hot corrosion behavior. The relatively continuous Al_2_O_3_ and TiO_2_ formed on K452 superalloy with higher content of Al and Ti have lower solubility and less damage in Na_2_O. Thus, the hot corrosion rate of K452 is lower than that of DZ40M with higher content of C, Cr, and W.

## 1. Introduction

Among the hot-end turbine blades of an aero-engine, those close to the combustion chamber are the guide vanes, i.e., the stationary blades. The blades convert the heat energy in the combustion chamber into pressure potential energy and transmit the airflow to the moving blades according to the required air inlet direction. This part of the blades is required to have good high-temperature strength, toughness, and other mechanical properties due to its harsh service environment, which is to work in high-temperature gas [1,2].

DZ40M and other cobalt-based superalloys have excellent high-temperature mechanical properties since more refractory metal elements W produce solid solution strengthening and more Cr, and C elements provide with carbide second phase strengthening [3,4]. Thus, they are often used as guide blade material, especially the first stage guide blades. However, due to the scarcity of cobalt resources, the price of cobalt-based superalloys is relatively high. Therefore, there is a tendency to replace cobalt-based superalloys with nickel-based superalloys. The nickel-based superalloys with good durability and creep properties are generally optimized by precipitation hardening. For example, the K452 with higher content of Al and Ti is strengthened by solid solution strengthening and dispersion strengthening of precipitated Ni_3_ (Al, Ti) [5,6], which is a typical precipitation hardening nickel-based superalloy with excellent performances at high temperatures. Thus, the K452 superalloy gradually replaces cobalt-based superalloys as turbine guide blades. Yet when the aircraft serve in ocean areas, the NaCl in the atmosphere would be sucked by the compressor along with the airflow. In addition, the NaCl attaches to the guide blades working in a high-temperature environment in the form of a molten salt film, accelerating blade corrosion and failure [7]. Finally, the mechanical properties, even as the service life of blades, could be significantly affected. Therefore, it is necessary to evaluate and analyze these two superalloys’ thermal corrosion failure behavior to provide a theoretical reference for the reasonable selection of guide vanes.

The hot corrosion of guide vane or working vane materials such as DZ40M and K452 has been widely studied. Part of the research aimed at the hot corrosion caused by Na_2_SO_4_ and NaVO_3_ molten salt, affecting inland gas turbine blades. The investigations believe that the equilibrium phase of Na_2_O and SO_3_ produced by Na_2_SO_4_ decomposition leads to alkaline or acidic dissolution of oxide film [8,9,10]. For example, D. Prabhakaran et al. [9] studied the hot corrosion behavior of SuperCo-605 in this environment and found that the oxidation products were mainly Cr_2_O_3_, MnO, NiO, etc. Hu et al. [10] studied the hot corrosion behavior of Ni-Cr coating in pure Na_2_SO_4_ molten salt. Another part of the research aimed at the hot corrosion caused by Na_2_SO_4_ and NaCl mixed molten salt, affecting the gas turbine blades working in coastal areas. For example, Fu. et al. [11] studied the hot corrosion of Co, Co-10Cr, and Co-10Cr-5Al in this environment; Masoumi. et al. [12] studied the hot corrosion of Hastelloy-X in this environment. The works proposed that Na_2_SO_4_ produced equilibrium phases of Na_2_O, O, and S in mixed molten salt, and Cl- was oxidized to Cl_2_. Thus, it is a synergistic mechanism of oxidation and vulcanization alkaline dissolution and chlorination to accelerate the destruction of oxide film [11,12,13,14].

Although there have been many explanations about the hot corrosion mechanism of pure Na_2_SO_4_, Na_2_SO_4_ and NaCl mixed molten salt on turbine blade materials, the topic aims at the hot corrosion of aircraft turbine guide blades in the high-altitude marine environment. Due to the high quality of aviation fuel and shallow sulfur content (<0.001%) [15], this situation can be regarded as the hot corrosion under the action of pure NaCl molten salt film. In addition, the hot corrosion behavior caused by Cl could be kind of different compared with those salts such as sodium sulphate (Na_2_SO_4_) and vanadium pentoxide (V_2_O_5_) However, the hot corrosion mechanism of this pure NaCl molten salt on materials has rarely been reported. Even though there are studies on the hot corrosion behavior of Superalloys under pure molten chlorine salt, they basically aimed at thermal energy storage equipment. In order to simulate the actual service environment, the material is soaked in molten salt, the salt supply is sufficient, and the O_2_ supply is limited [16,17,18], which can not truly reflect the hot corrosion of guide vanes.

In this work, DZ40M and K452 were selected to investigate the hot corrosion mechanism of NaCl molten salt at 900 °C. In addition, the two alloys’ corrosion behavior was compared and analyzed. The work could provide a theoretical basis for the follow-up blade protection measures and material selection application.

## 2. Materials and Methods

### 2.1. Materials

Cobalt-based superalloy DZ40M and nickel-based superalloy K452 were employed in this work, and their main constituent elements are shown in Table 1 and Table 2. A 15 mm × 10 mm × 2 mm sample was cut from the DZ40M substrate by wire cutting, and a semicircular sample with a radius of 8 mm was cut from the K452 substrate. The surface of the sample was polished with 240#, 400#, 800# sandpapers until it was smooth and chamfered step by step. In addition, the samples were cleaned ultrasonically in acetone and dried for later use.

**Figure 1:**
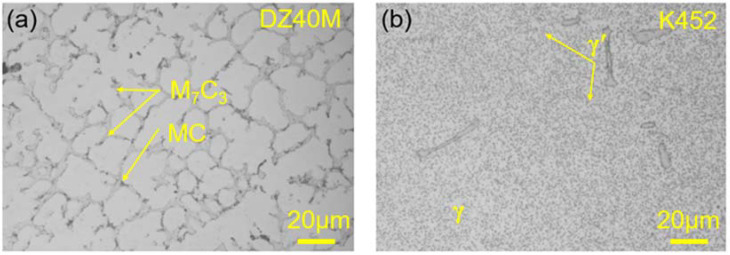
Microstructure of DZ40M and K452. (**a**) Metallographic photograph of DZ40M, (**b**) Metallographic photograph of K452.

Figure 1 shows the microstructures of DZ40M (a) and K452 (b). For DZ40M, in Figure 1a, under a metallurgical microscope, the Cr-rich M7C3 in the second phase is light-colored, showing coarse blocks, strips, and irregular shapes, while MC composed of W, Ti and other elements is black. In addition, for K452, in Figure 1b, the γ phase is the matrix phase composed of nickel-based solid solution; the γ’ phase is the dispersed precipitate phase composed of Ni_3_ (Al, Ti).

### 2.2. Isothermal Oxidation and Corrosion Exposure Test

**Figure 2:**
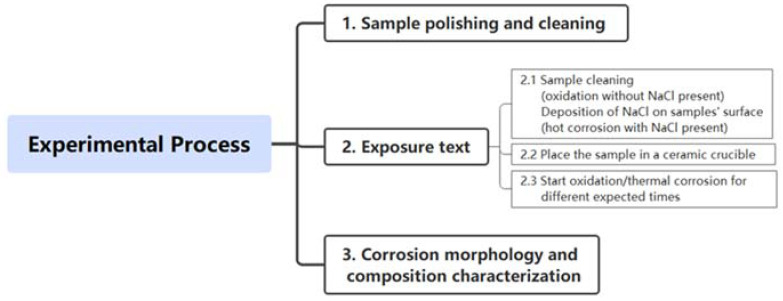
The scheme of experiment process.

For the investigation of alloys oxidized at 900 °C, the samples were placed in a crucible in a box muffle furnace at 900 °C for high-temperature oxidation experiments with atmospheres of air. The hot corrosion experiment was performed considering the influence of NaCl. To be introduced corrosive environments containing NaCl, the sample was first heated on an electric hot plate at 120 ~ 150 °C. Ultrafine droplets of mixed saturated NaCl solution and alcohol were sprayed to preheated specimens (~200 °C) by an ultrasonic atomizer. The ultrasonic atomizer’s operating frequency limited the droplet size to 20–40 m. In addition, a moderate operating frequency was chosen to control the droplet size so as to obtain pieces with a homogeneous and continuous NaCl deposit film on the single side of species. Then, samples with 4 mg/cm^2^ solid NaCl deposited were obtained by coating saturation NaCl solution on the heated samples’ surface. Finally, the NaCl-deposited samples were placed in a crucible and corroded in a 900 °C box-type muffle furnace. After oxidation and hot corrosion had been carried out for a period of time, the sample was taken out and air-cooled to room temperature. Then the oxidation and corrosion rate, product film composition, surface and cross-sectional morphology, and corrosion pit depth were characterized by kinetics, X-ray diffraction (XRD, X’Pert Pro Panalytica Co.), and energy-dispersive X-ray spectroscopy (EDS, INCA, X-Max), scanning electron microscope (SEM), roughness, respectively. The overall experimental process was shown in Figure 2.

## 3. Results

### 3.1. Oxidation and Corrosion Kinetics

**Figure 3:**
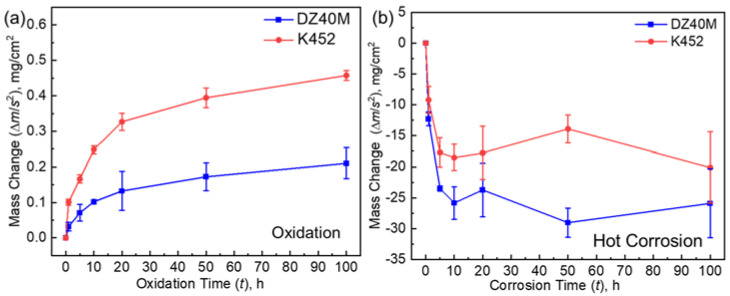
Kinetics curves of DZ40M and K452 at 900 °C. (**a**) High-temperature oxidation in air, and (**b**) hot corrosion with 4 mg/cm^2^ deposited.

Figure 3 displays the kinetics curves of DZ40M and K452 for high-temperature oxidation and thermal corrosion. Figure 3a,b is the kinetics curves of oxidation and hot corrosion, respectively. In Figure 3a, it can be seen that when the NaCl was absent, the oxidation weight gain of the two alloys was extremely slight at the same oxidation conditions. The oxidation kinetics basically satisfied the parabolic law. Further, the oxidation weight gain of K452 was nearly twice that of DZ40M during the whole exposure time, indicating that DZ40M was more resistant to oxidation. In Figure 3b, with the presence of NaCl at 900 °C, both alloys were severely corroded, resulting in significant weight loss. In this work, the salt was applied once before corrosion. Thus, In the early stage of thermal corrosion (0–10 h), salt was sufficient and thermal corrosion is more sufficient, and the corrosion products were seriously spalling, resulting in very large weight loss. In addition, the weight loss of DZ40M was significantly greater and more rapid than that of K452, indicating a poor heat-resistant corrosion performance of DZ40M. As the exposure time increased, the weight loss of the alloy gradually stabilized; still, the weight loss of DZ40M was greater than that of K452. However, NaCl was severely consumed during the thermal corrosion process, resulting in insignificant effect of residual NaCl on the thermal corrosion at the later stage (10–100 h). Meanwhile, due to fluctuations in the amount of spalling of the thermal corrosion products at each time node within 10–100 h, the weight changed occasionally increased. In addition, in both the kinetics curves shown in Figure 3a,b, as the exposure time increased beyond 10 h, the degree of their weight change decreased significantly for both the alloys, no matter the NaCl was present. A further comparing of Figure 3a,b also provide the influence of NaCl on the hot corrosion kinetics. As, can be seen, the weight change of hot corrosion was much greater than that of high-temperature oxidation. With NaCl absent, the weight change of both the alloys caused by oxidation process was below 0.5 mg/cm^2^, yet that of hot corrosion with NaCl present could greater than 10 mg/cm^2^, which proved the negative effect of NaCl on both the alloys during the thermal exposure. 

### 3.2. Macro-Morphology of Corroded Surface

**Figure 4:**
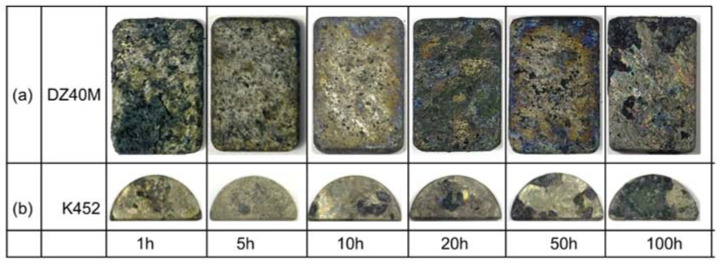
Macro-morphology of continuous hot corrosion of DZ40M and K452. (**a**) DZ40M, (**b**) K452.

Figure 4 displays the surface macro-morphology of continuous hot corrosion of DZ40M (a) and K452 (b) at 900 °C for 1 h, 5 h, 10 h, 20 h, 50 h, and 100 h. It can be seen that the corrosion was severe for both the alloys during all the exposure time. For DZ40M, as shown in Figure 4a, when the hot corrosion was performed for 1 h, the surface of the sample was covered by loose corrosion products. In addition, as the further increase of the exposure time, most of the corrosion products were directly peeled off from the bottom layer, and only a small part of the residual corrosion products was attached to the substrate. When the K452 underwent thermal corrosion at the same conditions, the corrosion products scale seemed to be better than that of DZ40M. However, the severe corrosion and spalling of corrosion products were also evident. Thus, it can be judged that the corrosion weight losses of both the alloys were obvious and can be regarded as the dominant factor, which went along with the results of corrosion kinetics shown in Figure 3b.

### 3.3. Oxidation and Corrosion Product Composition

**Figure 5:**
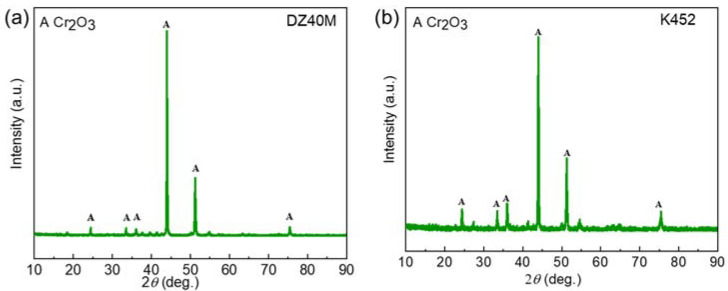
XRD of 100 h oxidation product of DZ40M (**a**), and K452 (**b**).

**Figure 6:**
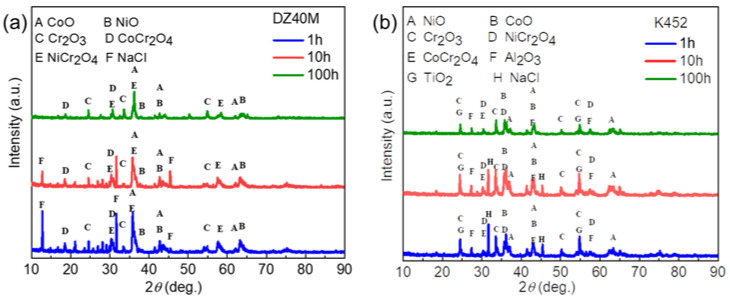
XRD of thermal corrosion product of DZ40M (**a**), and K452 (**b**).

Figure 5 is the XRD pattern of DZ40M (Figure 5a) and K452 (Figure 5b) after oxidation at 900 °C in air. It can be seen that the oxidation products produced by DZ40M and K452 were all Cr_2_O_3_. In addition, the Cr_2_O_3_ was the only product detected, which suggested the protection of Cr_2_O_3_. Figure 5 is the XRD pattern of DZ40M (Figure 6a) and K452 (Figure 6b) at hot corrosion conditions with NaCl absent at 900 °C. In Figure 5a, it was suggested that when NaCl was involved, the corrosion products of DZ40M mainly included CoO, NiO, CoCr_2_O_4_, NiCr_2_O_4_, and Cr_2_O_3_. In addition, the corrosion products of K452 (Figure 6b) mainly included CoO, NiO, CoCr_2_O_4_, NiCr_2_O_4_, Cr_2_O_3_, TiO_2_, Al_2_O_3_. In addition, after 1 and 10 h of hot corrosion, there was a certain amount of residual NaCl detected. When the reaction reached 100 h, the NaCl was exhausted, and XRD could not detect its presence. Comparing the XRD patterns shown in Figure 5 and Figure 6, it can be concluded that the NaCl destroyed the Cr_2_O_3_, which caused the faster inward diffusion of O and outward diffusion of metal elements, thus leading to the complex corrosion product composition and limited corrosion resistance.

### 3.4. Micro-Morphology and Composition of Surface

**Figure 7:**
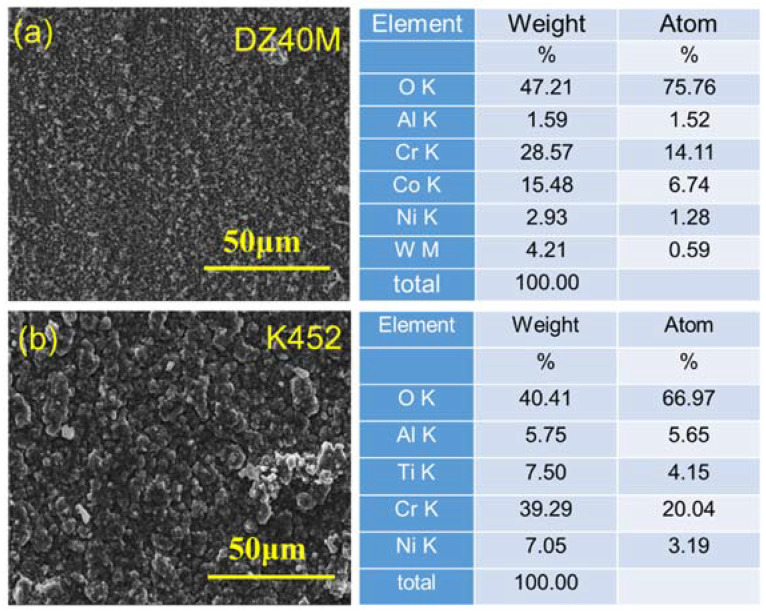
Surface morphology of DZ40M and K452 oxidized for 10 h and EDS. (**a**) DZ40M, (**b**) K452.

Figure 7 displays the micro-morphology and composition of oxidized DZ40M (a) and K452 (b). As observed in the surface morphology after 10 h oxidation at 900 °C in Figure 7a, the particles of the oxide film of DZ40M were relatively small, dense. In addition, according to the results of EDS, the oxidation scale was Cr_2_O_3_ doped with a small amount of Co oxide. While the particles of the oxide film of K452 (Figure 7b) were relatively coarse, with noticeable gaps between the grains, and the oxide contained a small amount of Ni oxide. Since the amounts of cobalt and nickel oxides were pretty low, XRD cannot detect them, but the EDS component analysis of microregion.

**Figure 8:**
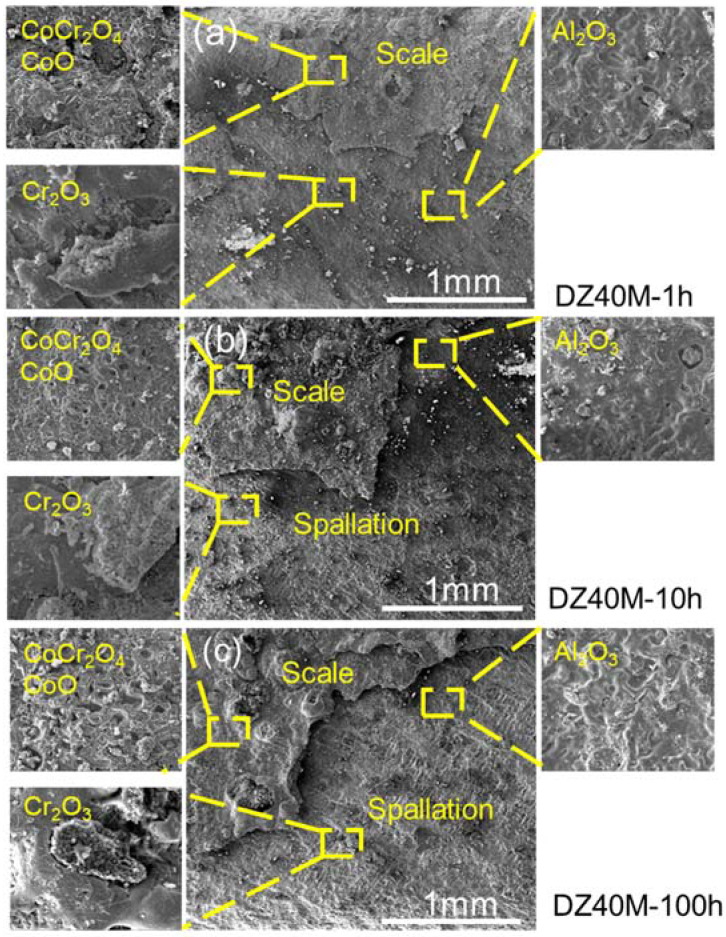
Surface morphology and composition of DZ40M after 1 h, 10 h, and 100 h of continuous hot corrosion. (**a**) 1 h, (**b**) 10 h, and (**c**) 100 h.

**Figure 9:**
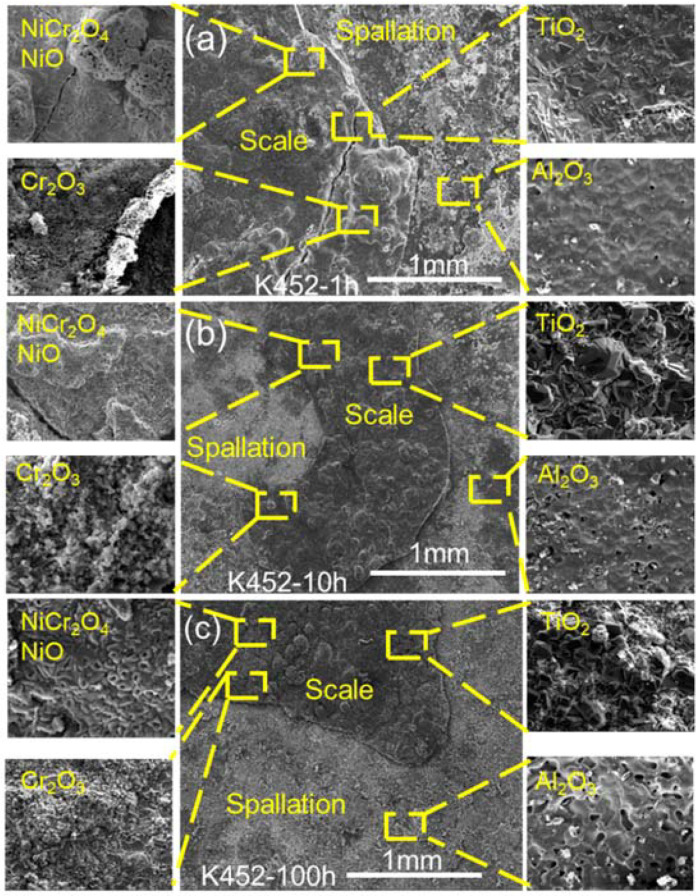
Surface morphology and composition of K452 after 1h, 10 h, and 100 h of continuous hot corrosion. (**a**) 1 h, (**b**) 10 h, and (**c**) 100 h.

When NaCl was involved, the surface morphology and composition of the alloys corroded for 1 h, 10 h, and 100 h was shown as Figure 8 and Figure 9. Due to the harsh corrosion environment, it seems more accurate to determine the corrosion process by comparing the initial short time (1 h) corrosion, the characteristic medium and long time (10 h) corrosion, and the long time (100 h) corrosion, as shown in Figure 8 and Figure 9, which avoided the error caused by the short sampling observation. Figure 8 is those of DZ40M, and it can be seen that after the corrosion of 1 h (Figure 8a), the scale was relatively completely with shallow crack. The micro area composition of the scale was summarized and shown in the enlarged images, which were CoCr_2_O_4_, CoO, Cr_2_O_3_, and Al_2_O_3_. As the increase of corrosion time to 10 h and 100 h, which were shown as Figure 8b,c, less difference of composition was detected, yet, the more obvious spallation was observed. The cracked scale consisted of CoCr_2_O_4_, CoO, which was the outmost scale, and the inner scale is composed of Cr_2_O_3_ and Al_2_O_3_.

Comparing with the surface morphology of DZ40M after corrosion shown in Figure 8, that of K452 corrosion for all the time range shown in Figure 9a–c were smoother. However, the spallation of the scale was still obviously observed. The main components of the outer layer oxide of K452, obtained by EDS analysis, were shown in the enlarged images in Figure 9: NiO, NiCr_2_O_4_, and Cr_2_O_3_, which were in the structure of spinel and arranged tightly locally. Yet, with the effect of NaCl, the Cr_2_O_3_ produced by the two alloys was severely damaged to be extremely loose and porous during the hot corrosion process. Thus, the scale had holes and cracks. In addition, the inner layer scale, which was composed of TiO_2_ and Al_2_O_3_ produced by the K452 hot corrosion, was denser but tiny holes. In addition, comparing the surface of oxidation alloys without NaCl present (Figure 7) and that of alloys suffered hot corrosion with NaCl present (Figure 8 and Figure 9), the present of NaCl led to a poorer surface morphologies with spallation and crack of the scale.

### 3.5. Cross-Sectional Morphology and Composition

**Figure 10:**
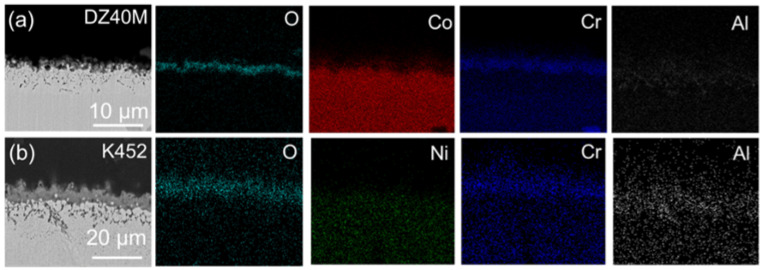
Cross-sectional morphology and EDS-mapping of DZ40M and K452 oxidation for 10 h. (**a**) DZ40M, (**b**) K452.

**Figure 11:**
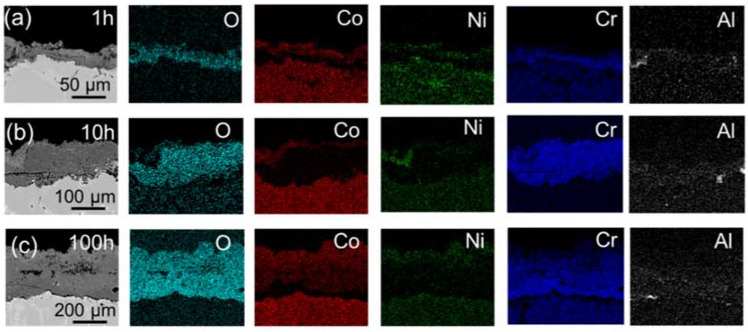
Cross-section morphology and mapping of DZ40M continuous corroded for 1 h, 10 h, and 100 h with NaCl present. (**a**) 1 h, (**b**) 10 h, and (**c**) 100 h.

**Figure 12:**
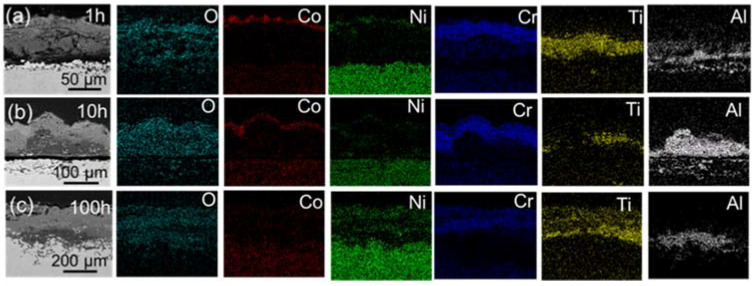
Cross-section morphology and mapping of K452 continuous corroded for 1 h, 10 h, and 100 h with NaCl present. (**a**) 1 h, (**b**) 10 h, and (**c**) 100 h.

Figure 10 displays the cross-section morphology and element distribution of DZ40M and K452 oxidized for 10 h. In addition, Figure 11 and Figure 12 are those of DZ40M and K452 corroded for 1 h, 10 h, and 100 h with NaCl present. It can be seen in Figure 10a that when NaCl was absent, the Cr_2_O_3_ layer produced by DZ40M was thinner after 10 h of oxidation, which was relatively consistent with the oxidation kinetics results. In addition, according to the distribution of Al, there were inner oxidation scale and internal oxidation zone composed of Al, which usually was Al_2_O_3_. In addition, in Figure 10b, the same distribution of Cr and Al was detected, which indicated the same structure of oxidation scale of K452 as that of DZ40M.

When NaCl was involved, as displayed in Figure 11 and Figure 12, it can be seen from the cross-sectional morphology and element distribution in Figure 11 and Figure 12 that after the hot corrosion performed for 1 h, 10 h, 100 h, DZ40M and K452 had formed thicker CoO, NiO, CoCr_2_O_4_, NiCr_2_O_4_ mixed oxide layer in the outermost layer, in which CoCr_2_O_4_ and NiCr_2_O_4_ may be formed by the solid-phase reaction of CoO and NiO with Cr_2_O_3_, respectively. Below this mixed oxide layer was a very thick Cr_2_O_3_ layer, which was much thicker than that formed by oxidation. The results were consistent with the surface morphology and composition shown in Figure 8 and Figure 9. Furthermore, under the Cr_2_O_3_ layer, DZ40M did not have other oxide layers, while K452 had successive TiO_2_ and Al_2_O_3_ layers, which were relatively continuous, and more severe Al internal oxidation occurred in the matrix. By comparing of the cross-section of alloys suffered oxidation without NaCl (Figure 10) and hot corrosion process with NaCl present (Figure 11 and Figure 12), the increasement of scales’ thickness with the influence of NaCl could be noticed, indicating the serious corrosion process induced by NaCl. In addition, it can be seen that an obvious internal corrosion occurred with O diffused inward and reacted with substrate, leading to the form of Cr_2_O_3_.

### 3.6. Roughness

**Figure 13:**
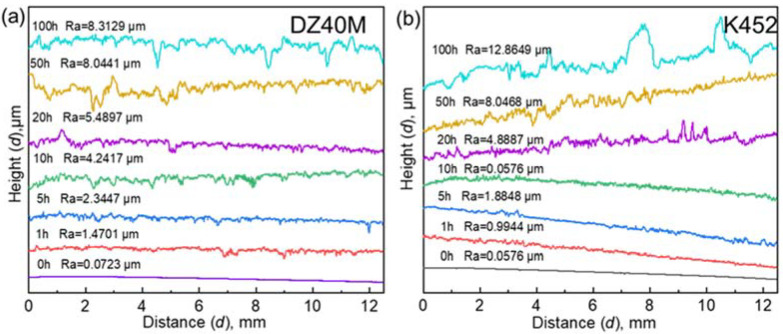
Substrate roughness of DZ40M and K452 after hot corrosion. (**a**) DZ40M, (**b**) K452.

**Figure 14:**
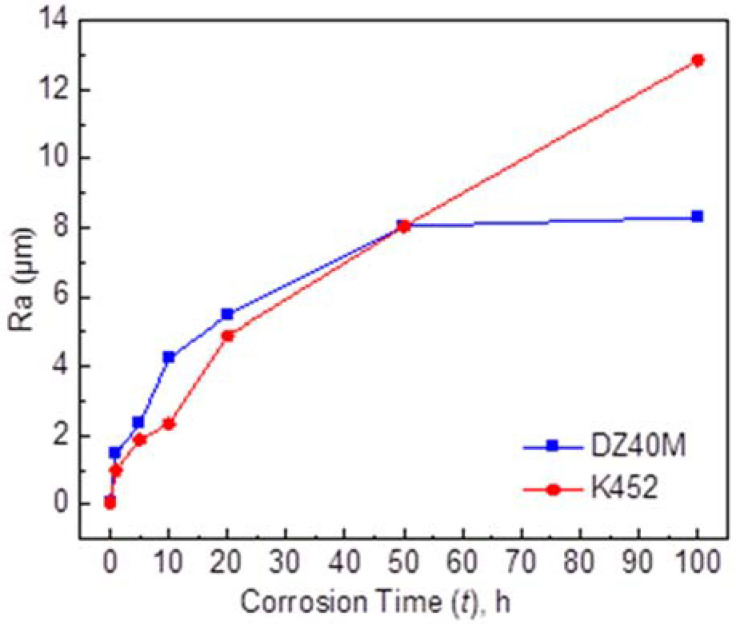
The substrate roughness changes of DZ40M and K452 after hot corrosion.

Roughness tests are performed after the removal of corrosion products. It was employed to measure the damage degree of alloy matrix under corrosion conditions, that is, the damage caused by internal corrosion. Figure 13a,b were the roughness of DZ40M and K452, respectively. In addition, Figure 14 summarized the change of roughness and their variation with time. The results reflected the surface roughness of the two alloys after hot corrosion and further suggested the tends of internal corrosion. As suggested by Figure 13 and Figure 14, during shorter corrosion time, which was no more than 10 h, more server severe roughness deterioration was detected for DZ40M. However, as the further extension of time, the roughness of K452 was more server. In addition, when the hot corrosion time reached 50 h, the roughness change value of DZ40M was obviously greater than that of K452, indicating serious internal corrosion of K452. Comparable, after 50 h, especially after a longer period of corrosion, the roughness change value of DZ40M tended to be stable, while the surface roughness of K452 deteriorated sharply. The degree of change far exceeded that of DZ40M. The situation may be caused by the greater content of Al and Ti for K452, which can react with O at an even low oxygen partial pressure, and thus leading to serious internal corrosion.

## 4. Discussion

### 4.1. Mechanism of NaCl-Induced Hot Corrosion 

#### 4.1.1. Alkaline Dissolution of Na_2_O

According to the kinetics results shown in Figure 1a,b, the deterioration of NaCl was greater and lead to the serious hot corrosion. By comparing the phenomena of oxidation shown in Figure 6a,b and hot corrosion shown in Figure 7, Figure 8, Figure 9, Figure 10 and Figure 11, it can be known that the Cr_2_O_3_ layer during the oxidation process was dense, yet, that was loose and porous after hot corrosion, which was destroyed by molten salt (NaCl). Combined with the hot corrosion theory discovered by previous researchers, Garces et al. [19] believed that NaCl can react with oxygen at high temperatures to form the alkaline equilibrium phase Na_2_O. Wei. et al. [20], when studying the hot corrosion of Mo-62Si-5B alloy in Na_2_SO_4_ + NaCl at 900 ℃, believed that the role of Cl element was to promote the dissolution of oxide film in coordination with O element, Yang. When they studied the thermal corrosion of Ni-Al coating in Na_2_SO_4_ + NaCl at 900 ℃, Yang. et al. [21] believed that Cl was oxidized first, and then reacted with metal elements in the form of Cl_2_ to generate gaseous chloride, which diffused outward and then was oxidized, increasing the internal holes of the oxide film and accelerating the oxidation. Accordingly, we can judge that sufficient NaCl reacts with O_2_. Thus, it could be proposed that the whole corrosion process occurred under the synergistic action of NaCl and O_2_. The influence of NaCl was first proposed to be the reaction of NaCl and O_2_ by which Na_2_O formed and destroyed the Cr_2_O_3_ scale, as described in Formulas (1) and (2). Then the Cl_2_ could transport inward and react with metal, thus, volatile metal chloride formed and diffused outward. Finally, the volatile metal chloride reacted with O_2_ and Cl_2_ was realized as described in Formula (3)–(5), and participated in the corrosion reaction again.

2NaCl + 1/2 O_2_→Na_2_O + Cl_2_↑ (1)

Na_2_O + Cr_2_O_3_→Na_2_Cr_2_O_4_ (2)

The Cl_2_ at the molten salt-oxide film interface enters the surface of the substrate through the Cr_2_O_3_ cracks and grain boundaries. It reacts with metal elements, as shown in Formulas (3)–(5), which causes the increase of Na_2_O activity at the molten salt-oxide film interface. Meanwhile, the Na_2_O activity gradient is formed in the molten salt, and the denser Cr_2_O_3_ begins to be alkaline dissolved by Na_2_O, as described in Formula (2).

Co + Cl_2_→CoCl_2_ (3)

Ni + Cl_2_→NiCl_2_ (4)

2Cr + 3Cl_2_→2CrCl_3_ (5)

As the outward diffusion of the metal chloride, especially the CrCl_3_, which has high reaction ability with oxygen, can be oxidized to Cr_2_O_3_. However, due to the Na_2_O activity gradient formation, the reaction between Cr_2_O_3_ and Na_2_O occurs again, which is described as Formula (2). On the other hand, the solubility gradient also provides the driving force for the diffusion of Na_2_Cr_2_O_4_ to the air-molten salt interface to cause the desolvation-reprecipitation in Formula (6). Finally, the loose and porous Cr_2_O_3_ is formed at the air-molten salt interface.

Na_2_Cr_2_O_4_→Na_2_O + Cr_2_O_3_ (6)

#### 4.1.2. The Role of Cl

The pores and defects formed in the corrosion film promote the inward transport of Cl_2_ and O_2_ in Formulas (2)–(4) and the outward diffusion and oxidation of gaseous chloride in Formulas (7)–(9). A large amount of CoO and NiO are generated in the outer layer of the corrosion product layer, and the thickness of Cr_2_O_3_ is also significantly increased.

CoCl_2_ + 1/2O_2_→CoO + Cl_2_↑ (7)

NiCl_2_ + 1/2O_2_→NiO + Cl_2_↑ (8)

2CrCl_3_ + 3/2O_2_→Cr_2_O_3_ + 3Cl_2_↑ (9)

The formed Cl_2_ (g) is proposed to react with metals as described in Formulas (3)–(5). During the hot corrosion process of K452, in addition to the above process, more Al and Ti elements are oxidized by O_2_ transported through the loose Cr_2_O_3_ to form more continuous Al_2_O_3_ and TiO_2_, which are relatively dense.

### 4.2. Differences in Hot Corrosion Behavior between DZ40M and K452

#### 4.2.1. The Effect of Higher Content of C, Cr, W in DZ40M

DZ40M had a faster hot corrosion rate, which may be due to the solid solution of the large amount of Cr in the matrix of DZ40M, and the second phase of the network is mainly composed of Cr_7_C_3_. During hot corrosion, the diffusion ability of Cr is great, and a large amount of Cr extends along grain boundaries and rapidly diffuses outward to participate in the oxidation. However, the generated Cr_2_O_3_ becomes loose and porous after being dissolved by Na_2_O, which cannot effectively prevent the outward diffusion of metal elements and the inward transmission of Cl_2_ and O_2_, leading to severe corrosion of DZ40M. At the same time, WC and other precipitates will block the outward diffusion of Ti and Al elements with lower content, and it is hard to form more continuous Al_2_O_3_ and TiO_2_.

#### 4.2.2. The Effect of Higher Content of Al and Ti in K452

K452 has better hot corrosion resistance, which may be due to its high Al and Ti elements content, which promotes the formation of thick and continuous Al_2_O_3_ and TiO_2_ during hot corrosion. It was considered that the solubility of Al_2_O_3_ was less than that of Cr_2_O_3_ in the alkaline equilibrium phase O^2−^. The same conclusion was also draw by Hu. et al. [10] when they studied the hot corrosion of Ni-Cr alloy in Na_2_SO_4_ and provided corresponding calculation of solubility. These two oxides, which are more resistant to alkaline Na_2_O dissolution than Cr_2_O_3_, have fewer holes and defects and can effectively prevent the outward diffusion of metal elements and the inward transport of Cl_2_ and O_2_. To a certain extent, they can protect K452 and make it suffer lighter hot corrosion. However, when NaCl is depleted in the later stage, the damage of the oxide film becomes slighter, the oxygen pressure at the interface of the substrate and corrosion film is low, and a large amount of Al is internally oxidized, which leads to a sharp deterioration of the flatness of the substrate. These phenomena can be seen in the cross-sectional morphology.

## 5. Conclusions

(1) NaCl and O_2_ form alkaline component Na_2_O at high temperature, which makes Cr_2_O_3_ loose and porous through the process of alkali solution, dissolution, and reprecipitation, leading to poor protection. Coupled with the cyclic effect of Cl, the structure and composition of the corrosion product film are changed, and the corrosion of the alloy is accelerated.
(2) DZ40M has a high C, Cr, and W elements content and generates plenty of loose and porous Cr_2_O_3_, which can not protect the matrix from severe hot corrosion.
(3) K452 produces Al_2_O_3_ and TiO_2_ with its high content of Al and Ti. It has a specific resistance to alkaline dissolution of Na_2_O, effectively preventing the diffusion of corrosive elements and lighter the degree of hot corrosion. However, due to a large amount of internal oxidation of Al in the later stage, the surface flatness deteriorates sharply, and the considerable depth of pitting pits causes stress concentration directly, which affects the mechanical properties of K452.

## Figures and Tables

**Table 1:** The main constituent elements and contents of DZ40M alloy (wt.%).

Elements	C	Cr	Ni	Co	W	Ti	Al
Contents	0.5	25.0	10.5	52.0	7.5	0.3	1.2

**Table 2:** The main constituent elements and contents of K452 alloy (wt.%).

Elements	C	Cr	Ni	Co	W	Ti	Al
Contents	0.11	21.0	56.5	11.2	3.5	3.5	2.5

## Data Availability

Not applicable.

## References

[1] Li F., Yang L., Zhou Y. (2019). Study Advances of High Temperature Coating for Aeroengine to resist Marine Atmospheric Corrosion. Therm. Spray Technol..

[2] Bu J., Gao Z., Han Z., Liu M., Niu J. (2020). Cracking Analysis of Low Pressure Turbine Guide Blade of Engine. Fail. Anal. Prev..

[3] Sun Z., Chen X., Zhang L., Zhang S., Feng J. (2021). Experimental and Numerical Study of Transient Liquid Phase Diffusion Bonded DZ40M Superalloys. Crystals.

[4] Duan C.Y., Liu P.S., Qing H.B. (2021). High temperature oxidation performance investigation on the activation energy of a Co-base superalloy oxidized in air. Mater. Lett..

[5] Li J., Yuan C., Guo J., Hou J., Zhou L. (2014). Effect of hot isostatic pressing on microstructure of cast gas-turbine vanes of K452 alloy. Prog. Nat. Sci. Mater. Int..

[6] Liu C., Peng J. (2015). Four hot corrosion resistant materials for IGT blades. Procedia Eng..

[7] Fan J., Liu G., Zhuo X., Zhang X., Feng J., Jiang W., Jiang Y., Yin J., He B., Hu Y. (2021). In-situ reaction synthesis Al_2_O_3_ overlay modified 7YSZ TBC for NaCl hot corrosion. Ceram. Int..

[8] Yu X., Song P., He X., Khan A., Huang T., Li C., Li Q., Lü K., Chen K., Lu J. (2019). Influence of the combined-effect of NaCl and Na_2_SO_4_ on the hot corrosion behaviour of aluminide coating on Ni-based alloys. J. Alloy. Compd..

[9] Prabhakaran D., Jegadeeswaran N., Somasundaram B., Raju B.S. (2020). Corrosion resistance by HVOF coating on gas turbine materials of cobalt based superalloy. Mater. Today Proc..

[10] Hu S., Finklea H., Liu X. (2021). A review on molten sulfate salts induced hot corrosion. J. Mater. Sci. Technol..

[11] Balashadehi M.M., Nourpour P., Aghdam A.S.R., Allahyarzadeh M.H., Heydarzadeh A., Hamdi M. (2020). The formation, microstructure and hot corrosion behaviour of slurry aluminide coating modified by Ni/Ni-Co electrodeposited layer on Ni-base superalloy. Surf. Coat. Technol..

[12] Fu G.Y., Zhao X., Liu Q., Su Y. (2011). Hot Corrosion of Cobalt-Based Alloy with (Na, K)_2_SO_4_ Coating at 900 °C. Adv. Mater. Res..

[13] Fu G., Li J., Liu Q. (2013). Hot Corrosion of Ni-based Alloys with Na_2_SO_4_ Coating at 900 °C. J. Shenyang Univ. Chem. Technol..

[14] Zhang J., Zhang Q., Zhuang Y., Kovalenko V., Yao J. (2021). Microstructures and cyclic hot corrosion behavior of laser deposited Inconel 718 alloy under different heat treatment conditions. Opt. Laser Technol..

[15] Mu M., Li J., Shen J., Sun Q., Wang Z. (2018). Analysis and research on quality of exported aviation kerosene. Refin. Chem. Ind..

[16] Cho S.H., Kwon S.C., Kim D.Y., Choi W.S., Kim Y.S., Lee J.H. (2019). Hot Corrosion Behaviour of Nickel-Cobalt-Based Alloys in a Lithium Molten Salt. Corros. Sci..

[17] Liu B., Wei X., Wang W., Lu J., Ding J. (2017). Corrosion behavior of Ni-based alloys in molten NaCl-CaCl_2_-MgCl_2_ eutectic salt for concentrating solar power. Sol. Energy Mater. Sol. Cells.

[18] Sun H., Wang J., Li Z., Zhang P., Su X. (2018). Corrosion behavior of 316SS and Ni-based alloys in a ternary NaCl-KCl-MgCl_2_ molten salt. Sol. Energy.

[19] Garces H.F., Tran A., Sternlicht H., Miller M., Resnick M., Marino S., Choi W.B., Padture N.P. (2020). Sea-salt-induced moderate-temperature degradation of thermally-sprayed MCrAlY bond-coats. Surf. Coat. Technol..

[20] Wei L., Shao W., Li M., Zhou C. (2019). Hot corrosion behaviour of Mo-62Si-5B (at. %) alloy in different molten salts at 900 °C. Corros. Sci..

[21] Yang Y.F., Liu Z.L., Ren P., Wang Q.W., Bao Z.B., Zhu S.L., Li W. (2020). Hot corrosion behavior of Pt+Hf co-modified NiAl coating in the mixed salt of Na2SO4-NaCl at 900 °C. Corros. Sci..

